# Metagenomic sequencing generates the whole genomes of porcine rotavirus A, C, and H from the United States

**DOI:** 10.1371/journal.pone.0244498

**Published:** 2020-12-29

**Authors:** Jennifer J. A. Hull, Mingpu Qi, Anna M. Montmayeur, Deepak Kumar, Daniel E. Velasquez, Sung-Sil Moon, Laura Cristal Magaña, Naga Betrapally, Terry Fei Fan Ng, Baoming Jiang, Douglas Marthaler

**Affiliations:** 1 Division of Viral Diseases, National Center for Immunization and Respiratory Diseases, Centers for Disease Control and Prevention, Atlanta, Georgia, United States of America; 2 State Key Laboratory of Agricultural Microbiology, College of Veterinary Medicine, Huazhong Agricultural University, Wuhan, Hubei, China; 3 Diagnostic Medicine and Pathobiology, Kansas State University, Manhattan, Kansas, United States of America; 4 Oak Ridge Institute for Science and Education, Oak Ridge, Tennessee, United States of America; 5 Department of Veterinary Population Medicine, University of Minnesota, St. Paul, Minnesota, United States of America; Arizona State University, UNITED STATES

## Abstract

The genus *Rotavirus* comprises eight species, designated *A* to *H*, and two recently identified tentative species *I* in dogs and *J* in bats. Species *Rotavirus A*, *B*, *C and H* (RVA, RVB, RVC and RVH) have been detected in humans and animals. While human and animal RVA are well characterized and defined, complete porcine genome sequences in the GenBank are limited compared to human strains. Here, we used a metagenomic approach to sequence the 11 segments of RVA, RVC and RVH strains from piglets in the United States (US) and explore the evolutionary relations of these RV species. Metagenomics identified *Astroviridae*, *Picornaviridae*, *Caliciviridae*, *Coronoviridae* in samples MN9.65 and OK5.68 while *Picobirnaviridae* and *Arteriviridae* were only identified in sample OK5.68. Whole genome sequencing and phylogenetic analyses identified multiple genotypes with the RVA of strain MN9.65 and OK5.68, with the genome constellation of G5/G9-P[7]/P[13]-I5/I5- R1/R1-C1-M1-A8-N1-T7-E1/E1-H1 and G5/G9-P[6]/P[7]-I5-R1/R1-C1-M1-A8-N1-T1/T7-E1/E1-H1, respectively. The RVA strains had a complex evolutionary relationship with other mammalian strains. The RVC strain OK5.68 had a genome constellation of G9-P[6]-I1-R1-C5-M6-A5-N1-T1-E1-H1, and shared an evolutionary relationship with porcine strains from the US. The RVH strains MN9.65 and OK5.68 had the genome constellation of G5-P1-I1-R1-C1-M1-A5-N1-T1-E4-H1 and G5-P1-I1-R1-C1-M1-A5-N1-T1-E1-H1, indicating multiple RVH genome constellations are circulating in the US. These findings allow us to understand the complexity of the enteric virome, develop improved screening methods for RVC and RVH strains, facilitate expanded rotavirus surveillance in pigs, and increase our understanding of the origin and evolution of rotavirus species.

## Introduction

Newborn piglets are susceptible to infections caused by numerous enteric microorganisms [1, 2]. Porcine rotavirus, discovered in 1970 [3], is a known enteropathogenic viruses associated with clinical diarrhea in a variety of animals, including humans. Belonging to the *Reoviridae* family, rotaviruses (RVs) have an approximate 18.5kb segmented, double-stranded RNA (dsRNA) genome in a non-enveloped, triple-layered icosahedral capsids [4]. The 11 dsRNA segments of the genome are distinguished by electrophoresis migration patterns in polyacrylamide gel electrophoresis (PAGE) and silver nitrate staining [5]. The viral genome encodes six structural (VP1-VP4, VP6 and VP7) and five or six nonstructural (NSP1-NSP5, and sometimes NSP6) proteins [4]. RVs are classified into eight species (A–H) based on antigenic properties and sequence based classification of the inner viral capsid protein VP6 [6]. A ninth group, RV group I in canine, in Hungary [7] and a tenth, RV group J in bats, in Serbia [8] have been identified and both proposed as rotavirus species. Reassortment between RV species has yet to be identified in nature.

Four of the ten RV groups (RVA, RVB, RVC, and RVH) have been detected in humans and pigs [4, 9], leading to the question of zoonotic transmission. RVA is the most prevalent and pathogenetic of the RV species. RVA was the leading cause of severe diarrhea among children until the human vaccines became available (Rotarix^®^ and RotaTeq^®^) [10–12]. Porcine RVA was isolated in 1975 [3] and are well known for their high prevalence and pathogenesis in pigs worldwide [13]. Reassortments between human and porcine strains have been well documented, and the Wa-like human backbone constellation shares a common ancestor with porcine [14, 15]. Due to the diversity of RVA strains, a binomial classification system is based upon serotype/genotype specificities and the sequence diversity of the two viral outer proteins, VP7 (glycosylated, G-genotype) and VP4 (protease-sensitive, P-genotype) [4]. To date, 36 G and 51 P RVA genotypes have been detected in animals, including humans, (https://rega.kuleuven.be/cev/viralmetagenomics/virus-classification/newgenotypes). Extension of the original VP7 and VP4 genotyping system created genome constellations with the complete description of Gx-P[x]-Ix-Rx-Cx-Mx-Ax-Nx-Tx-Ex-Hx, representing the genotypes of the genes VP7-VP4-VP6-VP1-VP2-VP3-NSP1-NSP2-NSP3-NSP4-NSP5/6, respectively, with x indicating the numbers of corresponding genotypes [16], and provisional whole-genome constellations have been established for RVB, RVC, and RVH [17–19].

Initially named pararotavirus, RVC was first detected in 1980 from a 27-day old piglet with diarrhea in Ohio [20, 21] and has been identified in cattle [22, 23], dogs [24], ferrets [25], mink [26], and in humans, mainly seen in children under age 3 years old [27, 28]. Identified as a major cause of disease in neonatal pigs [29], RVC has been detected in North and South America, Europe, and Asia [30–35]. Recently, the number of porcine RVC genomes have greatly increased and illustrate a higher genetic diversity of porcine RVCs compared to the other host species [36]. Generally, the RVC genotypes are host specific (bovine, canine, human, and porcine), excluding the VP6 and VP3 in which human and porcine strains share evolutionary relationship [36–38]. Originally, RVH was identified in humans from China and referred to as “adult diarrhea rotavirus” [ADRV-N] [39, 40] but later identified as RVH, based on serological and VP6 sequence analyses [6]. Subsequently, RVH has been detected in bats from Cameroon [41] and in pigs from Japan [9], Brazil [42], the United States (US) [43], and South Africa [44].

Infections with multiple RV species is common in swine. Studies have shown that infection with mixed RV species can intensify the severity of diarrhea in piglets [45, 46]. RVs cause direct economic losses to the pig industry, as neonatal diarrhea increases the morbidity and mortality rate, worldwide [47]. Next generation sequencing (NGS) is commonly used to generate viral genomes to understand the genotypic, evolutionary, and epidemiological relationship of multiple emerging and re-emerging porcine viruses [48–50]. We recently reported the nearly full-length RVH genome of strain RVH/Pig/wt/USA/MN9.65/2008/GxP[x] (MN9.65) from a pig in Minnesota, US [51]. In this follow-up study, we utilized the unbiased nature of viral metagenomics and the brute force of deep sequencing to examine the enteric virome of MN9.65 and another pig from Oklahoma (OK5.68) using next generation sequencing and report in-depth genetic relationship of RVA, RVC, and RVH, due to their recent emergence in swine.

## Materials and methods

A previous study identified RVH in 15% of piglet fecal samples [43]. Two positive samples, one each from Minnesota and Oklahoma (MN9.65 and OK5.68, respectively) were selected for metagenomics sequencing and sent to the Centers for Disease Control and Prevention (CDC). A previously described viral metagenomics approach was used to enrich and sequence viral nucleic acids [52]. Viral nucleic acid was amplified using a previously published sequence-independent, single-primer amplification (SISPA) protocol [52]. Amplicons were visualized on a TapeStation (Agilent Technologies, Santa Clara, CA), purified, and used for the NEBNext Ultra DNA Library Preparation Kit. The library was sequenced using the Illumina HiSeq platform with Rapid SBS Kit V2 (200 cycle).

Reads were analyzed using an in-house bioinformatics pipeline at the Division of Viral Diseases of the CDC [52–54]. After the virome analysis identified the viral taxa, in-depth manual analysis was performed using Geneious version 9.1.7 (Biomatters, Auckland, NZ) to generate the 11 RV segments.

### Genotype and phylogenetic analysis

Each RV gene segment was classified into genotypes based on previously described classifications [14, 17, 19]. Genotype specific sequence were downloaded from GenBank. Alignments were created using the CLUSTALW method in MEGA7 [55]. Genetic distances were calculated using the Kimura two-parameter correction at the nucleotide level using MEGA 7 [55]. Genotype specific phylogenetic trees were conducted using the Maximum Likelihood method with the General Time-Reversible nucleotide substitution model with 500 bootstrap replicates. For the RVA phylogenetic trees, preliminary trees were constructed, and clades lacking evolutionary relationship were removed to construct the final RVA trees illustrated in the manuscript.

### GenBank accession numbers

The 11 complete genome segments for RVA, RVC, and RVH strains from OK5.68 and the RVA from MN9.58 were deposited in GenBank while the previous RVH submission from MN9.58 was updated (Table 1).

**Table 1:** Genome constellation and GenBank accession number for each RV strain.

Strain Name	VP7	VP4	VP6	VP1	VP2	VP3	NSP1	NSP2	NSP3	NSP4	NSP5	Accession Numbers
RVA/Pig-wt/USA/MN9.65 /2008/G5/G9P[7]/[13]	G5/G9	P[7]/P[13]	I5/I5	R1/R1	C1	M1	A8	N1	T7	E1/E1	H1	MH267269-MH267284
RVA/Pig-wt/USA/OK5.68 /2008/G5/G9P[6]/[7]	G5/G9	P[6]/P[7]	I5	R1/R1	C1	M1	A8	N1	T1/T7	E1/E1	H1	MH308715-MH308731
RVC/Pig-wt/USA/OK5.68 /2008/G9P[6]	G9	P[6]	I1	R1	C5	M6	A5	N1	T1	E1	H1	MH282885-MH282895
RVH/Pig-wt/USA/MN9.65/2008/G5P[1]	G5	P1	I1	R1	C1	M1	A5	N1	T1	E4	H1	KU254582-KU254592
RVH/Pig-wt/USA/OK5.68 /2008/G5P[1]	G5	P1	I1	R1	C1	M1	A5	N1	T1	E1	H1	MH230116-MH230126

## Results

Samples were retrieved from young domesticated piglets with clinical signs of diarrhea, coughing and failure to thrive, during a study carried out by the University of Minnesota Veterinary Diagnostic Laboratory, US [43, 56]. The intestinal homogenates were sent to the Centers for Disease Control and Prevention (CDC) in Atlanta to generate the whole RVH genome. Using NGS, the viromes of the MN9.65 and OK5.68 were obtained, which generated 29,687,276 and 39,061,428 reads for MN9.65 and OK5.68, respectively, following quality trimming and filtering by our NGS analysis pipeline. While 21.76% and 23.05% of the reads from MN9.65 and OK5.68 were non-viral reads, 78.24% and 76.95% viral reads were generated, respectively. We also detected non-rotavirus viral pathogens, including *Astroviridae* (21.54% and 39.33%), *Picornaviridae* (4.93% and 33.23%), *Caliciviridae* (1.17% and 0.43%), *Coronoviridae* (0.13% and 1.21%) in MN9.65 and OK5.68, respectively. Additionally, *Arteriviridae* (0.14%) and *Picobirnaviridae* (0.02%) were observed in sample OK5.68. The in-depth virome analysis confirmed dual infection of RVA and RVH in sample MN9.65 and triple mixed infection of RVA, RVC and RVH in sample OK5.68. A majority of the reads (50.46%) in sample MN9.65 were Reoviridae and only 2.61% for OK5.68. Of the Reoviridae reads, the minor agent RVH accounted for 3.69% of the reads from MN9.65 and 21.69% from OK5.68. RVC only accounted for 12.34% of the Reoviridae reads in OK5.68. The RVA and RVH full genomes were obtained from MN9.65 and OK5.68 and RVC genome from OK5.68.

During the generation of the RVA gene segments for both samples, multiple sequences for the VP7, VP4, VP6, VP1, and NSP1 were determined, suggesting coinfection of RVA strains. The RVA strain MN9.65 contained two gene sequences in the VP7, VP4, VP6, VP1 and NSP4, yielding a mixed genome constellation of G5/G9-P[7]/P[13]-I5/I5- R1/R1-C1-M1-A8-N1-T7-E1/E1-H1 (Table 1). Also, the RVA strain OK5.68 contained two gene sequences in the VP7, VP4, VP1, NSP3 and NSP4, yielding a mixed genome constellation of G5/G9-P[6]/P[7]-I5-R1/R1-C1-M1-A8-N1-T1/T7-E1/E1-H1.

**Fig 1:**
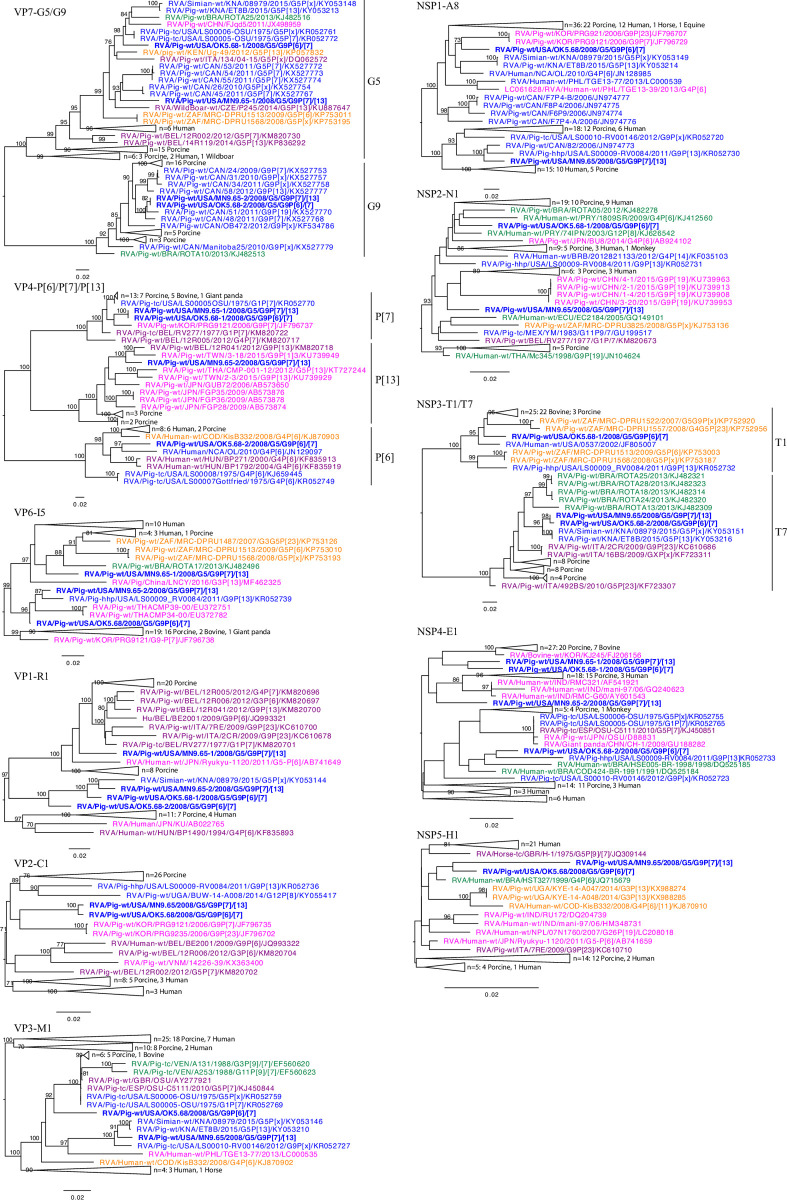
Phylogenetic trees of the RVA strains MN9.65 and OK5.68. The RVA strains from this study are represented in bold. The strains from Asia, North America, South America, Europe, and Africa are colored in pink, blue, green, purple, and orange, respectively.

The two G5 strains shared a 93% nucleotide identity and clustered within different branches of the phylogenetic tree and shared closer evolutionary relationship with porcine strains from Asia, and North and South America (Fig 1). The two G9 strains shared a higher nucleotide identity (98%) and clustered together within strains from Canada in the phylogenetic tree. Interestingly, the P[6] shared a common ancestor with a human strain from Nicaragua. The P[13] lacked evolutionary relationship with porcine strains from the North America and shared a common ancestor with strains from Taiwan and Belgium. The P[7] strains shared a 100% nucleotide percent identity and a common ancestor with a porcine Korean strain. In the trees for the remaining gene segments, the evolutionary relationship is complicated. For example, the VP6, NSP2, and NSP5 strains share a common ancestor with porcine and human strains, 3 of the 4 VP1 strains share a common ancestor with simian strain, and the VP3 and NSP3 strains share evolutionary relationship with bovine, human, porcine, and simian strains.

**Fig 2:**
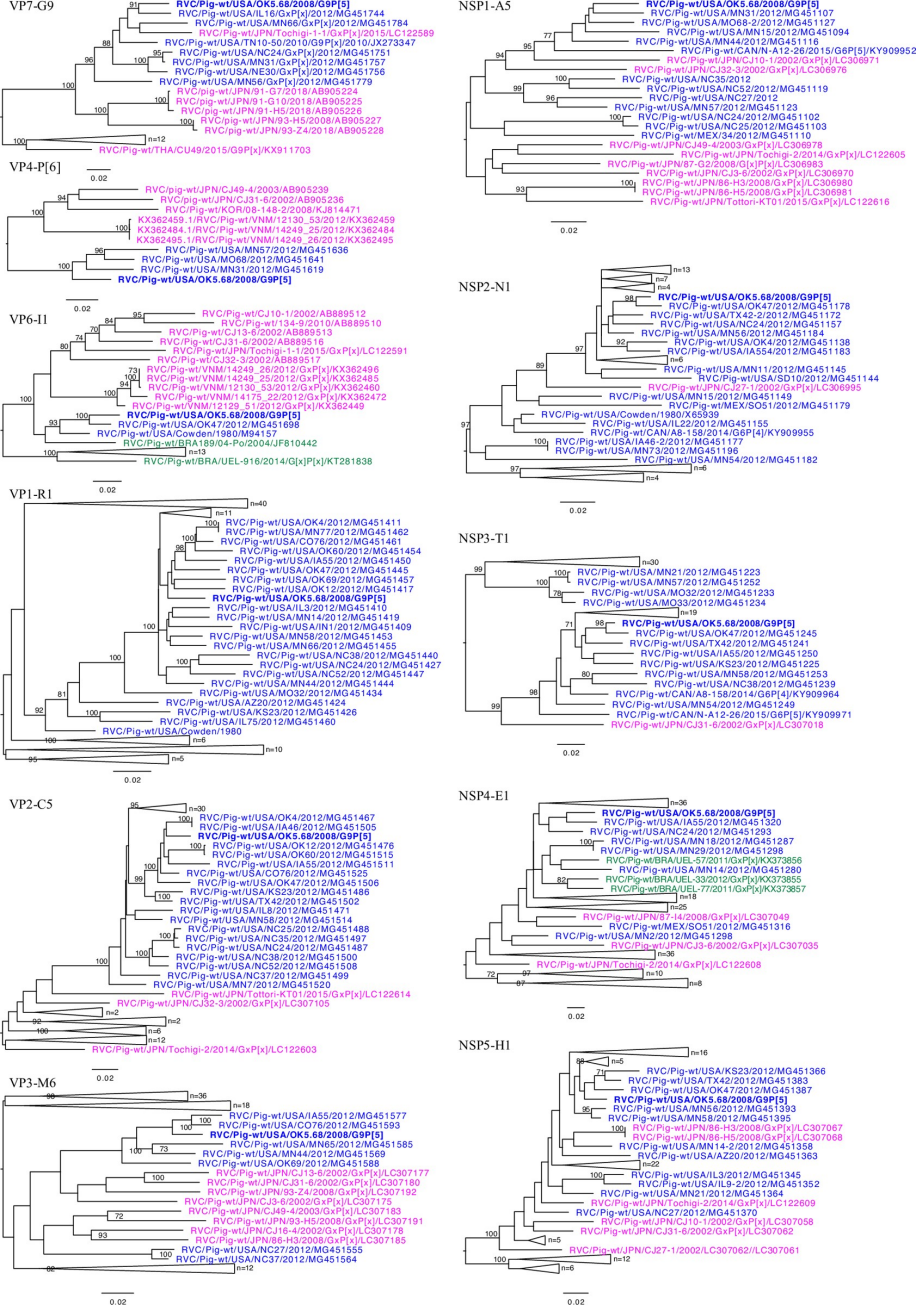
Phylogenetic trees of the RVC strain OK5.68. The RVC strain from this study is represented in bold. The strains from Asia, North America, and South America are colored pink, blue, and green, respectively.

Despite a very low percentages of RVC reads, the full-length nucleotide sequences for each gene was obtained, and mixed genotypes were lacking in the sample maybe due to the low number of RVC reads. Unlike RVA, in which the same genotype can be found within multiple host species, the RVC genotypes are host specific, excluding the VP6 and VP3 [17]. Multiple porcine genotypes exist for each RVC gene segment, and OK5.68 had a genome constellation of G9-P[6]-I1-R1-C5-M6-A5-N1-T1-E1-H1. The phylogenetic trees for each gene segment illustrated a most recent common ancestor to RVC strains from the US (Fig 2). While seven of OK5.68’s gene segments shared evolutionary relationship with strains from Oklahoma or Iowa, the VP7 and NSP1 genes was related to strains from Illinois and Minnesota, respectively. The US strains also shared a close evolutionary relationship with strains from Japan, and the VP4 gene from OK5.68 shared the closest evolutionary relationship with the Japanese strains.

**Fig 3:**
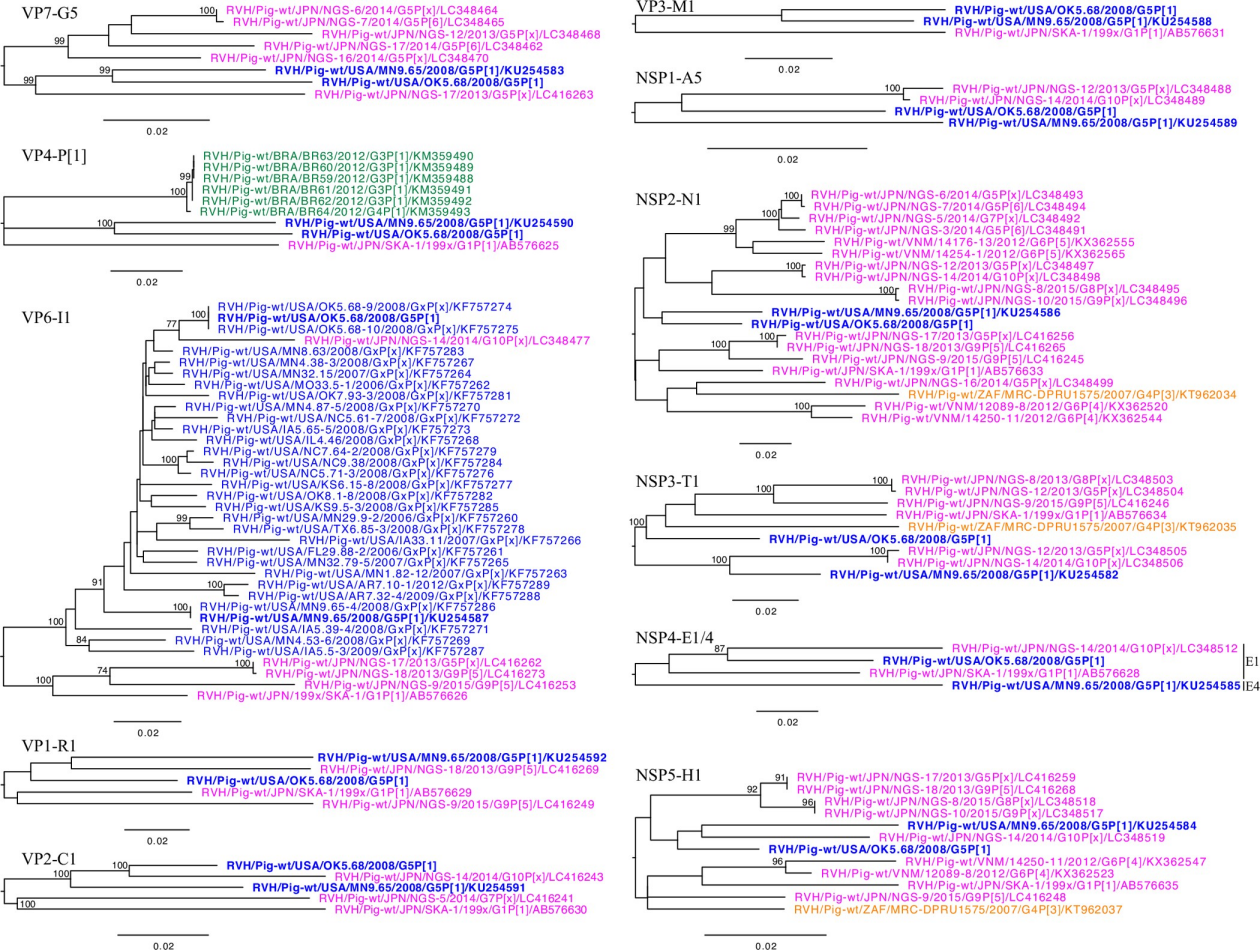
Phylogenetic trees of the RVH strains MN9.65 and OK5.68. The RVH strains from this study are represented in bold. The strains from Asia, North America, South America, and Africa are colored pink, blue, green, and orange respectively.

The provisional RVH genotype system identified host specific genotypes, and the human and bat strains have their own genotype constellations as expected [17, 41]. The multiple porcine genotypes have been identified in all the gene segments, except for the NSP2. The RVH genome constellation of MN9.65 and OK5.68 shared the same genome constellation of G5-P1-I1-R1-C1-M1-A5-N1-T1-H1, excluding the NSP4 genotypes (Table 1). The MN9.65 strain had an E4 genotype while OK5.68 had an E1 genotype. Of the eleven phylogenetic trees, our two RVH strains clustered together, illustrating distant evolution relationship from the Japanese strains, in the VP7, VP4, VP3, and NSP2 phylogenetic trees (Fig 3). Nevertheless, our RVH strains had evolutionary relation with the Japanese strain NGS-14 in the VP2, NSP1, NSP3, NSP4, and NSP5 phylogenetic trees. In the VP1 tree, our RVH strains clustered with Japanese strain NGS-18. These results indicate a complex evolutionary relationship for porcine RVH strains from the US and Japan.

## Discussion

Diarrhea is a common cause of mortality in piglets from enteric pathogens and may be due to a single agent, but concurrent infections are common [2]. Sequencing of the viral species in the sample was only possible with the use of NGS, which also identified other viruses in the samples [57]. Diagnostic methods, like polyacrylamide gel electrophoresis (PAGE) and enzyme-linked immunosorbent assay (ELISA) are known to be less sensitive compared to PCR and NGS. However, the vast diversity of the RV strains limits the detecting capacity of PCR. NGS analysis allowed for the positive identification and classification of our RV strains, in spite of the low viral load and the presence of mixed RV genotypes.

Morbidity and mortality associated with porcine RV infections varies depending on the virus strain, pig age, immunity status, and other concurrent enteric viral infections [58]. Porcine RV infection causes an increase mortality rate of 3–20% [59]. The use of the RVA vaccine to control and mitigate disease may have contributed a shift to RVC infections in neonatal piglets [29, 56]. The increased diversity in RVA strains could be due to the use of a porcine RVA vaccine in swine herds, the only federally approved vaccine incorporating G5 and G9 genotypes, which were identified in our samples. The continuous use of modified live virus vaccine in pigs may be directly contributing to the genetic diversity of RVAs via genetic reassortment between vaccine strains and wild type strain [13]. Such genetic rearrangements could also lead to the evolution of novel strains such as RVA G9 genotype. RVA G9 is a globally emerging genotype affecting humans and pigs and likely originating from pigs [60]. The lack of vaccine selective pressure and herd-immunity against the RVA G9 genotype probably explains its rapid emergence and spread in the pig population.

Unlikely RVA, the number of whole RVC genome data is limited. However, over the past few years, there has been an increased interest in human and porcine RVCs, and additional whole genome sequences have been published, which help elucidate their zoonotic relationship [17, 33, 35, 37, 61–67]. While a clade of VP3 segments of RVC strains from humans shared a common ancestor with porcine strains, Bayesian analysis illustrated these human and porcine strains diverged over a hundred years ago [36]. In the present study, only one sequence was detected in each segment, indicating the simple infection of RVC in sample OK5.68. In addition, our RVC strains shared the highest evolutionary relationship to US strains and distant relationship with Japanese strains. The VP6 gene segment shared evolutionary relationship with Brazilian strains, indicating our RVC strain is evolving with the American strains rather than the Asian strains. The lack of mixed RVC genotypes in the samples could either be due to the low RVC reads in the sample or due to the lack of RVC vaccine in pigs. Unlike RVA, a RVC vaccine is not available because the RVC strains are fastidious to grow in cell culture. The lack of vaccine selection pressure in RVCs might be the reason for less genetic diversity in the porcine strains. The limited number of global porcine RVC genomes hinders our knowledge on the evolutionary relationship of the internal gene segments. Additional porcine RVC sequences from Brazil and other countries would greatly elucidate the evolutionary relationship of porcine RVC strains.

Even though, the RV species share similar structural characteristics (11 segments of dsRNA and triple-layered capsid), pathogenesis and ability to cause disease is species specific. After weaning, co-infections with multiple RV species are common [46, 50], and porcine RVH has only been detected with RVA, RVB, or RVC, suggesting that porcine RVH is an opportunistic agent [9, 43, 44]. In this study, piglet diarrheal samples displayed this phenomenon, being co-infected with other RV species and other enteric viruses. In our phylogenetic analysis, both RVH strains MN9.65 and OK5.68 belonged to porcine constellations suggesting a lack of zoonotic transmission between human and porcine RVH strains. Interestingly, our two RVH strains had different genome constellations, indicating multiple porcine RVH constellations circulating within the US swine industry. However, additional sequencing of RVH strains from the US is needed to determine the diversity of RVH constellations circulating in the US.

This study provides analysis of the 11 complete segment sequences of the rare porcine RVH strains, and of RVA and RVC from the US. We fully assessed the viral diversity in both samples and were able to determine the complexity associated with multiple co-infection, and the presence of several variants within the two RVA strains. The evolutionary relationship of the RVA strains is complex while the RVC and RVH strains belong to porcine specific genotypes. The availability of complete genome sequences will allow for the development of new methods for RVC and RVH diagnosis and surveillance and determine the disease burden in pigs and humans in the United States and globally.
